# Development and Validation of the Schedule for the Assessment of Insight in Anxiety Disorders (SAI-A)

**DOI:** 10.1155/da/8843975

**Published:** 2025-09-24

**Authors:** Asala Halaj, Jonathan D. Huppert, George Konstantakopoulos, Anthony S. David

**Affiliations:** ^1^Department of Community Mental Health, University of Haifa, Haifa, Israel; ^2^Department of Psychology, The Hebrew University of Jerusalem, Jerusalem, Israel; ^3^First Department of Psychiatry, Medical School, National and Kapodistrian University of Athens, Athens, Greece; ^4^Research Department of Clinical, Education and Health Psychology, University College London, London, UK; ^5^UCL Institute of Mental Health, Division of Psychiatry, University College London, London, UK

**Keywords:** anxiety disorders, denial of mental disorder, illness awareness, insight into illness, need for treatment

## Abstract

There is a growing interest in understanding insight or illness awareness in anxiety; however, most assessment instruments were designed for psychosis. The unique features of anxiety highlight the need for tailored measures to accurately evaluate insight. The aim of this study was to develop and validate the Schedule for the Assessment of Insight in Anxiety (SAI-A), a clinician-rated scale for assessing insight in anxiety disorders. We interviewed 46 participants diagnosed with anxiety disorders, conducted SAI-A interviews, and administered self-report measures. Using correlation and principal component analysis (PCA), we identified and assessed scale components, ensuring their reliability and consistency. The SAI-A demonstrated acceptable psychometric properties, including convergent validity with an established self-report measure (*r* = −0.39, *p*=0.008) and internal consistency (Cronbach's α = 0.70). It showed moderate to strong agreement, interrater reliability (weighted kappa = 0.53, intraclass correlation coefficient [ICC] = 0.67), and test–retest reliability (ICC = 0.65). Two distinct insight components emerged: awareness of disorder and need for treatment. Higher overall SAI-A scores correlated with symptom severity and impairment (*r* = 0.56, *r* = 0.51, *p* < 0.001, respectively) and medication usage. The SAI-A is a valid and reliable assessment tool, providing a comprehensive framework for understanding and addressing insight in the context of anxiety disorders.

## 1. Introduction

Insight, the awareness and understanding of one's mental disorder or illness [1, 2], plays a significant role in characterizing various psychological disorders [3–7]. Insight has become a primary focus in psychosis research, as understanding it helps clarify the features and characteristics of the disorder [3]. Insight is recognized as a multidimensional construct, encompassing aspects such as awareness of illness, attribution of symptoms to the disorder, and recognition of the need for treatment [8]. The Schedule for the Assessment of Insight-Extended Version (SAI-E, [9]) is a widely used semi-structured interview for assessing insight in psychosis, covering symptom recognition, attribution, and understanding the need for treatment. It has been extensively studied, with research confirming its validity and reliability [10, 11].

More recently, there has been an expansion in the application of the SAI-E beyond psychosis. The SAI-E has been adapted for eating disorders (SAI-ED, [12]) and alcohol dependence (SAI-AD; [13]), demonstrating good psychometric properties. These adaptations aim to capture insight more effectively in these specific conditions, thus facilitating a better understanding of its role in these disorders.

While much research has focused on psychosis, an emerging area of interest is insight in anxiety using similar terms and concepts [1, 2, 5]. Despite the inconsistency in definitions of insight [5], studies have highlighted its association with symptom severity and treatment outcomes [5, 14–18], namely greater levels of insight were found to relate to increased symptom severity in both clinically diagnosed patients and individuals with high trait anxiety (the opposite pattern was shown in psychotic disorders). Ghaemi et al. [15] found a positive correlation between improved insight and better treatment outcomes in anxiety disorders. Vigne et al. [19] contributed to this area by suggesting an association between poor insight and nonadherence to cognitive–behavioral therapy. In specific phobia, insight is considered a cognitive bias, enhancing treatment planning and adherence predictions [14]. These findings emphasize the need for suitable bespoke tools to measure insight in anxiety [5].

Measures used to assess insight in anxiety disorders have often been adapted from instruments developed for psychosis. Examples include the Self-Appraisal of Illness Questionnaire (SAIQ; [20]) and the Schedule for Assessment of Insight (SAI; [1]), which have been used alongside the Diagnostic and Statistical Manual of Mental Disorders (DSM) insight criteria. These adaptations typically involve modifying the language to refer to anxiety or anxiety experiences instead of the original target conditions. Recognizing the limitations of existing measures, we developed the SAI in Anxiety (SAI-A). This assessment aims to capture insight's unique characteristics and address the complexities of assessing it in anxiety disorders [5, 18]. Current tools may not adequately address the interplay of cognitive, physical, and behavioral manifestations in anxiety. Moreover, certain characteristics of anxiety disorders, such as individuals perceiving their condition more as a “normal” psychological issue than a mental illness, and the complexity in recognizing when anxiety becomes excessive or starts affecting their lives (i.e., pathological), highlight the need for a tailored assessment tool. The SAI-A ensures a more accurate and comprehensive evaluation in addressing these issues. The purposes of this study were: (a) develop and validate the SAI-A in anxiety disorders, (b) assess the internal structure of the SAI-A and identify its components, and (c) explore the association between insight and anxiety symptoms and demographic features.

## 2. Methods

### 2.1. Construction of the SAI-A

The SAI-A is based on the SAI-E, which has demonstrated validity and reliability [10, 11]. In developing the content and structure of the SAI-A, we explored various dimensions of insight into anxiety, including awareness of symptoms and mental health condition/disorder, relabeling of symptoms, and acknowledgment of the need for treatment. To ensure clarity and relevance for anxiety, we chose terms like “mental disorder” or “psychological difficulties” over “illness,” as many participants who suffer from anxiety may associate “illness” more with more serious conditions. Additionally, we made efforts to comprehensively address elements related to anxiety, such as cognitions (e.g., believing that anxious thoughts are true), physical manifestations (e.g., increased heart rate, sweating, blushing, and their interpretation), and behavioral changes like avoidance. For relevance across different anxiety disorders, the scale includes examples as probes from the most common conditions, such as generalized anxiety disorder, social anxiety, specific phobias, and panic disorder.

The SAI-A items cover: (1) awareness of emotional or psychological difficulties, (2) awareness of anxiety and irrational fear or worries, (3) awareness of psychological or mental condition, (4) recognition of mental disorder, (5) attribution of condition to mental disorder, (6) awareness of adverse life consequences, (7) awareness of the need for treatment, (8) recognition of additional symptoms, (9) attribution of symptoms to mental disorder, (10) hypothetical contradiction (the individual's ability to consider another's perspective), and (11) treatment engagement. The formulation of the scale and the rating scores closely align with those adapted from the SAI-E in psychosis (see the SAI-A in Appendix 1). The total SAI-A score is calculated by summing the scores of all 11 items, with a possible range from 0 to 28. Higher total scores indicate higher levels of clinical insight into anxiety symptoms, including awareness of their psychological nature and the need for treatment.

### 2.2. Participants

The recruitment process involved public advertisements and inviting participants from the university recruitment pool. Out of the 70 invited participants, 46 agreed to participate in the study, meeting criteria for a score of 4 or higher on the Overall Anxiety Severity and Impairment Scale (OASIS; [21]) and demonstrating fluency in either Hebrew or Arabic. The final participant sample included 34 individuals fluent in Hebrew and 12 fluent in Arabic. Each group received the version of the assessment corresponding to their language proficiency.

### 2.3. Measures

Measures were translated into Hebrew or Arabic and back-translated into English for translation validity.

The SAIQ [20] is an 8-item self-report measure used to assess attitudes toward mental illness. It was modified to address anxiety, covering concerns about anxiety's impact on relationships and work, the belief in its potential improvement, along with the acknowledgment of the need for treatment and awareness of how thoughts affect daily life. Higher total scores indicate lower levels of clinical insight. The measure has acceptable psychometric properties [20]. In our sample, Cronbach's α was 0.73.

The Beck Cognitive Insight Scale (BCIS; [22]) is a 15-item self-report measure assessing cognitive insight. Higher composite scores indicate greater cognitive insight (higher self-reflectiveness). The scale showed good psychometric properties [23]. In our sample, Cronbach's α was 0.65.

The Insight Self-Report measure was specifically developed for this study to evaluate participants' perception of their anxiety and awareness of the need for treatment. This 10-item questionnaire utilizes a 4-point Likert scale, with participants responding to statements like “I consider my anxiety to be a psychological problem/illness or disorder/normal reaction,” “I believe my anxiety is caused by stress/chemical imbalance/an inherited condition/part of life or personality/related to childhood experiences,” and “The way to help me with my anxiety is psychological therapy/taking prescribed medication/talking to a friend/ignoring my anxiety/dieting and/or exercising.” Some items are reverse-scored (items 3, 4, and 6). Responses may reflect either insight into aspects of anxiety or, conversely, a tendency to minimize the condition by favoring self-help methods, dietary changes, or exercise as primary solutions. Higher scores on this scale indicate greater overall level of clinical insight, suggesting a more comprehensive understanding of one's anxiety condition, its psychological nature, and the potential need for professional help (psychological/psychiatric treatment). In our sample, Cronbach's α was 0.68. For reference, the measure is provided in Appendix 2.

The State–Trait Inventory for Cognitive and Somatic Anxiety (STICSA; [24]) is a 21-item measure of cognitive and somatic symptoms of anxiety. The trait subscale was used in this study, and higher scores indicate higher severity of symptoms. The measure has acceptable psychometric properties [25]. In our sample, Cronbach's α was 0.75.

The OASIS [21] assessed anxiety frequency, intensity, and functional impairment in the past week as well as being used to define study entry. Higher total scores indicate more severe anxiety-related impairment, with a clinical cut-off score of greater than 8. The scale showed good psychometric properties [21]. In our sample, Cronbach's α was 0.81.

### 2.4. Procedures

Participants provided written informed consent and underwent screening to assess study eligibility. Interested participants then were administered a structured interview, the Diagnostic Interview for DSM-5 Anxiety, Mood, and Obsessive–compulsive and related disorders (DIAMOND; [26]), followed by the administration of the SAI-A. Postinterview, participants completed a battery of online self-report measures, assessing insight and symptom severity in random order, all on the same day. For interrater reliability evaluation, 24 initial interviews were audiotaped and independently rated by two additional raters. Test–retest reliability was assessed by readministering the SAI-A to participants 1 week later by the same interviewer. Participants received compensation for their participation.

### 2.5. Statistical Analysis

Pearson correlation tests were used to examine relationships between individual items of the SAI-A and the total score. For convergent validity, Spearman correlation assessed the association between the SAI-A and insight measures. Internal consistency of the SAI-A and its subscales was evaluated using Cronbach's α. Test-retest reliability and interrater reliability were assessed through intraclass correlation coefficient (ICC). Principal component analysis (PCA) and multidimensional scaling (MDS) were performed to identify potential components of the SAI-A items. An eigenvalue of 1 was set as the criterion for factor retention in PCA. Further analysis of the association between insight degree, symptom severity, and clinical features was conducted through correlation analyses. Given that many variables did not adhere to a normal distribution, as indicated by the Kolmogorov test and kurtosis values, Spearman's coefficient correlation was applied for respective analyses. A significance level of *p*  < 0.05 was set for all statistical tests.

## 3. Results

### 3.1. Demographic Characteristics

Table 1 provides an overview of participant demographics. The age of participants ranged from 20 to 60 years, mean age = 28 years (SD = 8.24); 72% of participants were female. Out of the total participants, 8 (17%) reported using medication, 4 (8%) were currently in treatment, while 20 (43%) had undergone past treatment. Regarding anxiety diagnoses, 39 (84%) participants received a diagnosis of social anxiety disorder, 26 (56%) of generalized anxiety disorder, 8 (17%) of panic disorder, 7 (15%) of agoraphobia, 4 (8%) of specific phobia, and 1 (2%) participant received a diagnosis of separation anxiety.  Table 2 contains the means and standard deviations for all study measures assessed.

### 3.2. SAI-A Reliability and Validity

The SAI-A demonstrated acceptable internal consistency, with a Cronbach's α of 0.70. Alpha coefficients for each item, if deleted, ranged from 0.67 to 0.70. Item-total correlations varied from 0.23 (“recognition of additional symptoms”) to 0.68 (“awareness of psychological condition” and “attributions of symptoms to mental disorder”). Two items, “recognition of additional symptoms” and “treatment engagement,” showed weak correlations (*r* = 0.23 and *r* = 0.28) with the SAI-A total score (Appendix 3).

In terms of SAI-A convergent validity, the SAI-A total score showed an expected significant negative correlation with the SAIQ total score (*r* = −0.39; *p*=0.008). Divergent validity was supported by nonsignificant correlation with the BCIS total score [17]. Additionally, the SAI-A total score was correlated with the insight SR total score (*r* = 0.57; *p* < 0.0001), indicating a strong relationship (Table 3).

For test–retest reliability, there was a total score ICC value of 0.70, whereas ICC values ranged from 0.50 to 0.98 for individual items, indicating good reliability over time. Interrater reliability, assessed using ICC, demonstrated acceptable reliability for the overall score with an ICC value of 0.67, reflecting good consistency in the ratings. ICC interrater values per item ranged from 0.33 to 0.95, with three items (“awareness of psychological changes,” “recognition of additional symptoms,” and “treatment engagement”) showing excellent interrater reliability, with (ICC > 0.76). These findings demonstrate the strong interrater reliability of the SAI-A, both at the item level and for the overall assessment.

### 3.3. Internal Structure of SAI-A

**Figure 1:**
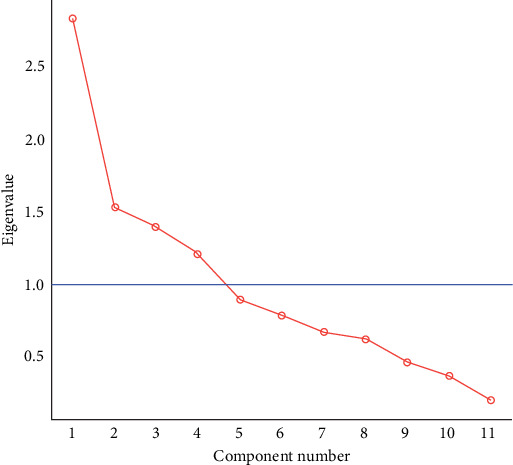
Screen plot of factors extracted from the principal component analysis of the SAI-A.

**Figure 2:**
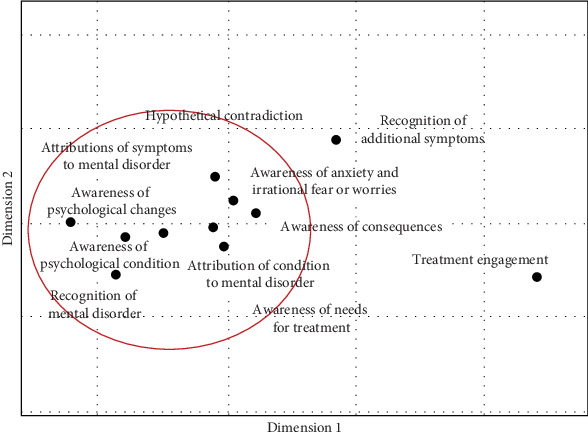
Plot from multidimensional scaling analysis of the items of the Schedule for the Assessment of Insight in Anxiety (SAI-A).

To assess sample adequacy for PCA, we conducted the Kaiser–Meyer–Olkin (KMO) test. The initial overall KMO value was 0.56, just above the minimum threshold of 0.5. Items 8, 10, and 11 showed particularly low individual KMO values. While removing these items would have improved the overall KMO to 0.68, we decided to retain all items because the SAI-A is an interview-based measure, and each item provides useful clinical information. We then conducted a PCA on the SAI-A to explore its internal structure using all items, interpreting results cautiously given the potential limitations in factor structure reliability. In the initial analysis with unconstrained factors, we observed two main underlying themes: one related to awareness of disorder/symptoms, and the other associated with treatment beliefs and engagement. Visual representation of the extracted components is presented in Figure 1. This shows the first principal component to explain considerably more of the variance than the second to fourth components, which have similar eigenvalues. Considering this observation, we reran the PCA, specifying two factors. The refined analysis revealed that the first factor explains 25.72% of the variance and includes items related to awareness of psychological changes (SAI-A item 1), psychological condition (SAI-A item 3), recognition of mental disorder (SAI-A item 4), and attributions of symptoms to a mental disorder (SAI-A item 9). Factor 2 explains 39.62% of the variance and includes items related to the need for treatment (SAI-A item 7) and treatment engagement (SAI-A item 11). Additional details and specific loadings for each item are outlined in Table 4. In addition to the PCA, we conducted MDS to further validate the results and explore the underlying structure of the SAI data. The MDS analysis showed two-dimensional structure accounted for 41.13% of the total variance. Dimension 1 ranged from “treatment engagement” at one extreme to “attributions of symptoms to mental disorder” at the opposite extreme, with “Recognition of additional symptoms” and “awareness of consequences” also loaded on this dimension. Dimension 2 was characterized by “recognition of additional symptoms” and ‘Hypothetical contradiction' at one end, with “awareness of need for treatment” and “recognition of mental disorder” at the opposite end. Most items clustered closely together, while “treatment engagement” and “recognition of additional symptoms” were positioned farther from the main cluster, and to a lesser extent, “awareness of need for treatment” was also somewhat distinct (Figure 2).

### 3.4. Association Between Insight and Demographic Characteristics

The relationship between SAI-A total score and using medication was found to be significant (*ρ* = 0.30, *p*=0.043), suggesting better insight in those who took medication. However, no significant correlation was observed between SAI-A total score and other demographic characteristics, including age (*ρ* = −0.02, *p*=.875), gender (*ρ* = −0.08, *p*=0.571), marital status (*ρ* = −0.16, *p*=0.295), education (*ρ* = 0.20, *p*=0.182), employment (*ρ* = 0.27, *p*=0.067), and past treatment (*ρ* = 0.093, *p*=0.537).

### 3.5. Association Among Insight and Anxiety Measures

The SAI-A total score showed a significant positive correlation with STICSA (*ρ* = 0.55, *p* < 0.001) and a similar positive correlation with OASIS (*ρ* = 0.52, *p* < 0.001). These findings suggest that higher levels of insight were associated with more severe anxiety symptoms and greater impairment (Table 3).

## 4. Discussion

This study aimed to develop and validate the SAI-A, while examining the relationships between insight levels, anxiety symptoms, and demographic characteristics in anxiety disorders. Higher overall SAI-A scores correlated with symptom severity and impairment. SAI-A total score correlated with medication usage but not with other demographic characteristics. This study makes a significant contribution by introducing the SAI-A, a new clinical rating assessment for measuring insight in anxiety disorders. This addresses a gap identified by a recent review by Halaj and Huppert [5], which highlighted the lack of measures for assessing insight in anxiety.

The results confirm the reliability and validity of the SAI-A as a measurement tool. The good internal consistency enhances the instrument's reliability. While the internal consistency (Cronbach's α = 0.70) is at the lower end of the acceptable range, it is comparable to previous adaptations of the SAI, such as the version for alcohol use disorder [13]. This result likely reflects the complexity and multidimensional nature of insight, as well as the heterogeneity of the construct. Future refinements, such as clarifying item wording and scoring criteria, may help to improve internal consistency. Furthermore, item-total correlations contribute to our understanding of the individual items' impact on the overall score.

While most items demonstrated satisfactory associations, two items, namely “recognition of additional symptoms” and “treatment engagement,” exhibited weaker correlations. This weaker correlation can likely be attributed to the nature of the “recognition of additional symptoms” item. While the first two questions seem related to cognitive insight, as they address beliefs and thoughts, the item includes emotional and behavioral aspects as well. Therefore, it offers a broader perspective beyond cognitive insight alone. The individual variability in perception and response could contribute to the observed weaker correlation. Exploring these variations through additional participant feedback would be helpful. Clinicians might also consider asking more detailed questions to better differentiate participants' responses, ensuring a more accurate reflection of their unique experiences related to each symptom. Despite its lower correlation, the “recognition of additional symptoms” item is important to retain. Insight assessment involves both general awareness of having a disorder and specific recognition of individual symptoms. This item provides valuable information about particular anxiety symptoms, complementing the measure of overall anxiety awareness. This comprehensive approach enhances the assessment's depth, allowing for a more nuanced understanding of the individual's condition and potentially improving intervention strategies.

The weaker correlation of the “treatment engagement” item with the total SAI-A score may be due to its comprehensive nature, including a broad spectrum of potential treatment modalities, including medication use, therapy sessions, and seeking informational resources. Participants might express reservations about specific treatments, leading to a less direct alignment with their overall level of awareness of the need for treatment. This tallies with research suggesting that individuals with anxiety prefer not to use medication, despite being aware of their symptoms [27]. Also, the decision to engage in treatment might involve various factors beyond disorder awareness, such as beliefs about treatment effectiveness, unique preferences, past experiences, and cultural considerations like stigma. Some participants might express reservations or uncertainties about specific treatment methods mentioned in the item, leading to a less direct alignment with their overall insight level. Nevertheless, including these items in the scale ensures that it covers variations around insight in anxiety, which would be missed with a single- or fewer-item scale. Convergent validity was established, as evidenced by negative correlations with the SAIQ. This negative correlation is expected, as higher scores on the SAI-A reflect higher levels of insight, whereas higher scores on the SAIQ reflect lower levels of insight. The absence of a significant correlation with the BCIS total score suggests that the SAI-A captures a distinct dimension. This finding was not unexpected, considering that the BCIS primarily pertains to a cognitive style entailing the questioning of beliefs and perceptions (including “unusual experiences”) rather than disorder awareness. This difference raises questions about the relationship between cognitive and clinical insight. The complexity of this relationship is reflected in prior findings in anxiety, where research found low or no correlation [16, 17]. Further exploration will provide details on the nature of this relationship in anxiety disorders.

Test–retest reliability analysis confirmed the stability of SAI-A scores over time, showing significant correlations between scores at two time points. Interrater reliability indicated a moderate level of agreement between raters, reflecting consistency in their assessments. The moderate to high ICC values confirm the reliability of this measurement, both for the overall score and individual items. The strong ICC score for the SAI-A total score further assures its dependability. Furthermore, when examining ICC values for individual items, we observed strong agreement among raters for most items. These results emphasize the SAI-A's consistency, whether considering the whole assessment or item by item. Such reliability is essential for the overall utility of the SAI-A. Further research is needed to examine the properties of the scale when examining change, such as in treatment and longitudinal studies. Two items, “attribution of condition to mental disorder” and “hypothetical contradiction," exhibited low ICC values. For the “attribution of condition to mental disorder” item, this variability likely reflects the complexity of these items and differing interpretations among raters, particularly in distinguishing which aspects or events the patient responses referred to (e.g., personality traits, life stress, or long-standing habits). These discrepancies suggest a need for additional clarification on possible patient responses. Similar challenges have been reported in other adapted insight measures. For example, Konstantakopoulos et al. [12] found attribution-related items in the SAI-ED difficult to rate consistently due to variability in patient interpretations and cognitive rigidity, suggesting that such variability may be expected when using subjective, interpretive items across clinical groups. To improve interrater reliability, future applications could benefit from detailed scoring guidelines, example responses, structured follow-up questions, and rewording to clarify the item's intent. Despite the low ICC values, this item remains important as it provides information about patients' self-perception and their understanding of how the disorder impacts their lives. Retaining it ensures a more accurate assessment, which is essential for effective treatment planning and intervention. For example, recognizing that patients attribute their psychological difficulties to external factors rather than their condition can highlight areas where they might need further education or support.

Regarding the “hypothetical contradiction” item, individuals experiencing anxiety typically have a clear understanding of their feelings and thoughts, eliminating the need for external validation. This contrasts with psychosis, where individuals may lack contact with reality and require reassurance or struggle with perceiving facts, often due to paranoia. While the abstract nature of this item may contribute to rater differences, incorporating structured probes or clarifying the expected range of patient responses could improve consistency. Despite initial considerations of removing this item, the subsequent analysis revealed that its exclusion did not result in significant improvements. This observation suggests that the item may hold value as it prompts a consideration of hypothetical scenarios and aids in recognizing their feelings and thoughts that might otherwise be avoided or denied. Given its role in promoting self-reflection and revealing emotional and cognitive aspects, we believe retaining this item is necessary to ensure a comprehensive evaluation of insight.

The PCA revealed two primary factors that collectively explain a substantial 65.3% of the variance, each contributing to the overall structure of the scale. Factor 1 reveals a notable association with items related to awareness of symptoms and mental/psychological disorder. Factor 2 focuses on treatment beliefs, more specifically, awareness of the need for treatment and treatment engagement. These findings confirm that insight into anxiety disorders encompasses multiple dimensions. The results from both PCA and MDS provide support for a two-dimensional structure. While the specific variance explained differs between PCA and MDS, both analyses suggest a two-dimensional conceptualization of insight in anxiety disorders as measured by the SAI-A. This consistency across different analytical methods strengthens our confidence in the multidimensional nature of anxiety insight.

The MDS map suggests a two-dimensional structure of insight. Dimension 1 seems to represent a continuum from recognizing symptoms to attribution to a mental disorder. Dimension 2 contrasts the ability to recognize additional symptoms and engage in hypothetical thinking with recognition of having a mental disorder and recognizing the need for treatment. This dimension might reflect the complexity of insight, ranging from fundamental awareness to greater depth of understanding of one's condition

The MDS map shows that treatment engagement and recognition of additional symptoms were positioned far from the main cluster of items, and to a lesser extent, awareness of the need for treatment was also somewhat distinct. This pattern is consistent with the lower item–total correlations observed for these items, suggesting that they reflect more specific and context-dependent aspects of insight that do not align as closely with the core aspects of the construct. For example, treatment engagement may be influenced by prior experiences with treatment, trust in medical professionals, and cultural attitudes toward seeking help rather than by awareness of illness alone. In addition, the vague wording of this item, referring to “any professional” such as a family doctor, psychologist, or psychiatrist without specifying the type of professional, may also contribute to its distinct position. Similarly, recognition of additional symptoms (i.e., distorted beliefs, physical symptoms of anxiety, avoidance behaviors) may reflect a higher degree of self-reflection and introspective awareness, representing a more advanced understanding of one's condition that is not equally present in all individuals. Although these items show weaker psychometric alignment with the overall scale, they provide clinically meaningful information and were therefore retained.

Regarding the association between insight and anxiety symptoms, our findings reveal positive correlations between the SAI-A total score and both STICSA and OASIS scores. This indicates that higher symptom severity leads to increased awareness of difficulties and suffering, acknowledging the presence of a problem or disorder. This observation is consistent with previous studies by Halaj and Huppert [17] and Halaj et al. [16] in both clinically diagnosed patients and individuals with trait anxiety. It is important to note that this association may reflect bidirectional influences: individuals with higher insight may report their symptoms more accurately, or the experience of more severe symptoms may in turn enhance awareness and insight into the disorder. Given that the anxiety measures were administered by self-report, the current study could not determine whether lower anxiety was due to minimizing or denial of symptoms or due to their truly being lower symptoms. A previous study demonstrated that the discrepancy between clinician rating and self-report was a significant predictor of clinical insight in panic disorder [16], suggesting that the former rationale is likely to be at least part of the picture. The other possibility, that lower symptoms lead individuals to believe less that they have a disorder and that it needs treatment, is a reasonable stance for mild anxiety disorders in that it avoids over-medicalization, in contrast to other disorders such as psychosis. An important implication of our results is the potential for tailored interventions targeting individuals with moderate to severe pathological anxiety who lack insight. By emphasizing how insight significantly influences their daily lives, interventions could effectively enhance their awareness of the broader impact of anxiety, motivating them to seek appropriate treatment.

Regarding the relationship between insight and other characteristics, a significant correlation between the SAI-A total score and medication usage was found. This suggests that individuals with higher levels of insight are more likely to use medications. This is not surprising, given that insight is related to awareness regarding treatment-seeking, and individuals taking medication have sought treatment. No substantial correlations were detected between the SAI-A total score and demographic factors, including age, gender, marital status, education, and employment. These findings stress the independence of insight from these demographic variables, again adding to the practical utility of the schedule.

The SAI-A is promising for both research and clinical use. In research, it offers a detailed assessment of insight specific to anxiety experiences. This allows researchers to explore how insight relates to treatment outcomes, symptom severity, and anxiety changes over time, as well as its relationship to other aspects of anxiety disorders. Clinically, the SAI-A helps practitioners understand patients' perspectives on their difficulties. This understanding can improve treatment planning, help target suitable interventions and enhance clinician–patient communication. Overall, this measure can improve our understanding of insight and potentially lead to better strategies and interventions in both research and clinical settings.

This study has several limitations. While we used self-report measures for convergent validity, the SAI-A relies on clinical interviews, providing a more comprehensive assessment of insight and its dimensions. Although self-report measures are efficient, they may not capture all aspects. Considering the benefits of clinical interviews despite higher costs could strengthen convergent validity evidence. Future research needs to incorporate both help-seeking and nonhelp seeking samples, especially those with more severe conditions such as agoraphobia, to confirm its wider applicability. Additionally, the relatively modest sample size for the PCA and the limited diagnostic diversity of our sample warrant caution in interpreting the factor structure. Replication of these findings in larger and more diagnostically diverse samples is essential to confirm and refine the scale's dimensional structure. Finally, the limited socioeconomic and geographic diversity of the sample may limit the generalizability of the findings. Future research should examine the psychometric properties and associations of the SAI-A in more socioeconomically and geographically diverse populations to ensure its broader applicability.

## 5. Conclusions

Our study introduces the SAI-A as a reliable and valid tool for assessing insight in anxiety disorders. The independence of insight from demographic factors highlights its universal relevance, while analyses of the SAI-A's psychometric properties confirm its reliability and validity. The observed associations between insight and anxiety symptoms provide valuable findings into the complex relationship between disorder awareness and the intensity of anxiety experiences. This new assessment tool bridges existing research gaps and offers a tailored approach to assessing insight, with the goal of improving clinical understanding for those facing these challenges.

## Figures and Tables

**Table 1:** Participants demographics.

Demographics	*N*	Percent	Mean	SD
Age	46	—	28	8.24
Sex				
Male	13	28	—	—
Female	33	72	—	—
Martial status				
Single	39	85	—	—
Married	7	15	—	—
Type of education				
High school	8	17	—	—
Bachelor's	31	67	—	—
Master's/doctoral	7	16	—	—
Years of education	—	—	13.5	1.70
Employment				
Employed	29	63	—	—
Unemployed	17	37	—	—
Treatment status				
Present treatment	4	0.08	—	—
Past treatment	20	43	—	—
Medication				
Medication yes	8	17	—	—
Medication no	38	83	—	—
Alcohol consumption	21	46	—	—
Anxiety diagnosis				
Social anxiety	39	84	—	—
Panic	8	17	—	—
Agoraphobia	7	15	—	—
Generalized anxiety	26	56	—	—
Specific phobia	4	8	—	—
Separation anxiety	1	2	—	—
Total	46	100	—	—

**Table 2:** Means and standard deviations of study measures.

Measure	Mean (SD)
SAI-A	15.2 (4.05)
BCIS	3.32 (4.54)
SAIQ	10.5 (3.37)
Insight SR	26.06 (4.47)
STICSA	49.65 (12.28)
OASIS	7.67 (4.80)

Abbreviations: BCIS, beck cognitive insight scale; Insight SR, insight self-report; OASIS, overall anxiety severity and impairment; SAI-A, schedule of assessment of insight; SAIQ, self-appraisal of illness questionnaire; STICSA, state–trait inventory for cognitive and somatic anxiety.

**Table 3:** Spearman coefficient correlations between insight and symptoms severity measures.

Variables	SAI-A	BCIS	SAIQ	Insight SR	STICSA	OASIS
SAI-A	1	—	—	—	—	—
BCIS	0.16	1	—	—	—	—
SAIQ	−0.38*⁣*^*∗*^	−.34*⁣*^*∗*^	1	—	—	—
Insight SR	0.57*⁣*^*∗*^	0.07	−0.30*⁣*^*∗*^	1	—	—
STICSA	0.55*⁣*^*∗*^	0.15	−0.41*⁣*^*∗*^	0.37*⁣*^*∗*^	1	—
OASIS	0.52*⁣*^*∗*^	−0.03	−0.35*⁣*^*∗*^	0.13	0.63*⁣*^*∗*^	1

Abbreviations: BCIS, beck cognitive insight scale; Insight SR, insight self-report; OASIS, overall anxiety severity and impairment scale; SAI-A, schedule of assessment of insight; SAIQ, self-appraisal of illness questionnaire; STICSA, state–trait inventory for cognitive and somatic anxiety.

*⁣* ^*∗*^Significantly different from zero at *p* < 0.05.

**Table 4:** Principal component loading of the SAI-A.

SAI-A items	PCA factor	
	1	2
1. Awareness of psychological changes	**0.44**	−0.11
2. Awareness of anxiety and irrational fear or worries	0.28	−0.42
3. Awareness of psychological condition	**0.47**	0.03
4. Recognition of mental disorder	**0.44**	0.04
5. Attribution of condition to mental disorder	0.17	−0.04
6. Awareness of consequences	0.30	0.17
7. Awareness of needs for treatment	0.24	**0.54**
8. Recognition of additional symptoms	−.06	−0.03
9. Attributions of symptoms to mental disorder	**0.37**	0.00
10. Hypothetical contradiction	0.06	**−0.55**
11. Treatment engagement	−0.01	**0.43**
Percentage of variance explained	25.7%	39.6%

*Note:* Bold values indicate factor loadings ≥|0.37|.

## Data Availability

The data are available from the corresponding author upon reasonable request.

## Supporting Information

Supporting Information Appendix 1 contains the full Schedule for the Assessment of Insight in Anxiety (SAI-A). Appendix 2 includes the Insight Self-Report measure developed for this study. Appendix 3: Internal Consistency, interrater and test–retest reliability of SAI-A items.
