# Supplementary material for: Development and Validation of the Schedule for the Assessment of Insight in Anxiety Disorders (SAI-A)
Source: Depress Anxiety. 2025 Sep 24;2025:8843975. doi: 10.1155/da/8843975 (PMC12488311; doi:10.1155/da/8843975)
Supplement: Supporting Information — Appendix 1 contains the full Schedule for the Assessment of Insight in Anxiety (SAI-A). Appendix 2 includes the Insight Self-Report measure developed for this study. Appendix 3: Internal Consistency, interrater and test–retest reliability of SAI-A items. [file 8843975.f1.docx]

**Appendix 1: ­ Schedule for the Assessment of Insight in Anxiety (SAI-A)**

| 1. “Do you think you have been experiencing any emotional or psychological difficulties?”  *If the answer is yes, then ask the patient, “What are these emotional or psychological difficulties?”*  *(Refer to these changes and difficulties when asking about item 4 and item 7.)*  *If the answer is no, ask the patient “Do you think you have been experiencing anxiety, fear, or*  *worry?”*    *often* (thought present most of the day, most days) = 2  *sometimes* (thought present occasionally) = 1  *never* = 0 |
| --- |
| If brief write verbatim reply, otherwise summarise response. Please add explanatory comments if appropriate. |

| 2. “Do you think that you have anxiety, fears, or worries that are more than the average person or irrational?"    *Yes* (sure it’s irrational/more than the average person) = 2  *Unsure* (cannot decide if it’s rational/ more than the average person) = 1  *No* = 0 |
| --- |
| If brief write verbatim reply, otherwise summarise response. Please add explanatory comments if appropriate. |

| 3. “Do you think this means there is something wrong psychologically or mentally?” (For example, a nervous or stress disorder/condition).  *If previous answer was “never” or “no” ask; “If others think you have been experiencing emotional /psychological changes/ difficulties, do you think there must be something wrong even though you do not feel it yourself?”*  *often* (thought present most of the day, most days) = 2  *sometime*s (thought present occasionally) = 1  *never* (ask why others think so [if the answer to the question of others is yes]) = 0 |
| --- |
| If brief write verbatim reply, otherwise summarise response. Please add explanatory comments if appropriate. |

If positive score on previous two items, proceed to 4, otherwise go to item 6.

| 4. “Do you think your condition amounts to a mental disorder/condition *(use patient’s terms based on patient’s previous answers mentioned in item 1*)?”  *If the patient is not clear about the meaning of mental disorder, discuss in terms of a problem or difficulty.*  *often* (thought present most of the day, most days) = 2  *sometimes* (thought present occasionally) = 1  *never*  = 0 |
| --- |
| If brief write verbatim reply, otherwise summarise response. Please add explanatory comments if appropriate. |

If positive score on items 1,2, and 3, proceed to 5, otherwise go to item 6.

| 5. “How do you explain your anxiety, fear or worry /disorder/condition?” *(use patient’s terms)*  *Reasonable account given based on plausible mechanisms*  (appropriate given social, cultural and educational background,  e.g. excess stress, chemical imbalance, family history, part of personality*,* etc.) = 2  *Confused account, or overheard explanation without adequate*  *understanding or “don’t know”* = 1  *Other explanation (e.g., I am a weak person, part, I am lazy, I am bored)* = 0 |
| --- |
| If brief write verbatim reply, otherwise summarise response. Please add explanatory comments if appropriate. |

| 6. “Has your nervous/emotional /psychological /mental disorder/condition or problem *(use patient’s term)* led to adverse consequences or problems in your life? (For example, conflict with others, financial or accommodation difficulties, poor work performance, problems at home or in social settings, and irrational, impulsive or dangerous behaviour).  *Yes* (with example) = 2  *Unsure* (cannot give example or contradicts self) = 1  *No* = 0 |
| --- |
| If brief write verbatim reply, otherwise summarise response. Please add explanatory comments if appropriate. |

| 7. “Do you think your ... disorder/condition (*use patient’s term)* or the problem resulting from it warrants (needs) treatment?”  *Yes* (with plausible reason) = 2  *Unsure* (cannot give example or contradicts self) = 1  *No* = 0 |
| --- |
| If brief write verbatim reply, otherwise summarise response. Please add explanatory comments if appropriate. |

| 8. Pick the most prominent symptoms up to a maximum of 4. Then rate awareness of each symptom out of 4 as below  Examples:  a. “Do you think your beliefs/fears are true?”  *Panic disorder*  - The belief that you are having a panic attack  - The belief that if you have a panic attack you will die, faint or have a heart attack  - The belief that you will lose control or “go crazy” during a panic attack  *Social anxiety disorder*  - The belief that people think you are inadequate/worthless  - The belief that others will reject you  - The belief that you are going to do something embarrassing or say something stupid  *Generalized anxiety disorder*  - The belief that you have no control over your worry  - The belief/fear that you will lose your mind because of worrying  - The belief/fear that something will happen to you/your friend/or family member  *Specific phobia*  -The belief that if you see a dog it will run over you and bite you  -The belief that in small places you wont be able to get out and will lose control or fain  -The belief that in medical procedures/blood injury you will faint or that the pain will be  unreasonable  -The belief that in heights, you will fall or be killed  b. “Do you think experiencing physical symptoms (For example, excessive sweating, dizziness, nausea, muscle tension, racing or increased heart rate, shortness of breath) means something catastrophic will happen to you?”  c. “Do you avoid people, places, situations, or activities because you are anxious/distressed?”  “ Do you you think that bad things will happen to you if you do not avoid places, situations or activities?  “ Do you think that your fear is unbearable?”  d. “Do you feel anxious or fearful without good reason?”  Symptom 1 - type: Symptom 2 - type: Symptom 3 - type: Symptom 4 - type:  rating rating rating rating    *Definitely* (full awareness) = 4  *Probably* (moderate awareness) = 3  *Unsure* (sometimes yes, sometimes no) = 2  *Possibly* (slight awareness) = 1 mean  *Absolutely not* (no awareness) = 0 |
| --- |
| If brief write verbatim replies, otherwise summarise responses. Please add explanatory comments if appropriate. |

| 9. For each symptom rated above (up to a maximum of 4), ask the patient ... “How do you explain ... your beliefs, physical symptoms; or behaviors *(select the most suitable examples based on the patient’s previous answers mentioned in item 8)*  Symptom 1 Symptom 2 Symptom 3 Symptom 4    *_Due to nervous condition/disorder_* _= 2 mean_    *Unsure, don’t know/can’t say* = 1  *Other explanation (e.g., personal weakness, part of life)* = 0 |
| --- |
| If brief write verbatim reply, otherwise summarise response. Please add explanatory comments if appropriate. |

| 10. “How do you feel when people do not believe you? For example, when you say one of the following (*pick the most suitable example based on the patient’s aforementioned symptoms or difficulties mentioned in item 8*):   1. Panic Disorder   “My heart is racing, and I might be having a heart attack.”  If someone says you will not have a heart attack it is just anxiety, how do you feel?  Do you feel like they do not believe you? How do you feel when they do not believe you?   1. Social anxiety disorder   “If I speak in front of people or make a mistake, they will laugh at me and think I am  stupid.”  If someone says this belief is not true, how do you feel? Do you feel like they do not believe you? How do you feel when they do not believe you?   1. Generalized anxiety disorder   “I haven’t heard from my friend. Something must have happened to them.”  If someone says nothing has happened to your friend (even if they don’t really know), how do you feel?  Do you feel like they do not believe you? How do you feel when they do not believe you?  “I worry a lot and I cannot control it, other people do not worry too much”  If someone says you should be able to control your worries, how do you feel?  Do you feel like they do not believe you? How do you feel when they do not believe you?   1. Specific phobia   “If the dog/spider bites me, then the bite might kill me.”  If someone says the bite will not kill you (even if they don’t really know), how do you feel?  Do you feel like they do not believe you? How do you feel when they do not believe you?  *I know that I have a problem or disorder* = 4  *I wonder whether something’s wrong with me or whether I have a problem* = 3  *I’m confused and I don’t know what to think* = 2  *I’m still sure despite what others say* = 1  *They’re lying* = 0 |
| --- |
| If brief write verbatim reply, otherwise summarise response. Please add explanatory comments if appropriate. |

| 11. “If a therapist or psychiatrist/doctor recommended that you should seek treatment, how likely would you be to comply?  *If the patient asks what is meant by treatment, give examples: taking some medications, doing therapy, reading some information.*  *Very likely* = 4  *Likely* = 3  *Natural* = 2  *Not likely =1*  *Very unlikely = 0* |
| --- |

Schedule for the Assessment of Insight (SAI-A)

| Patient’s Name |  |
| --- | --- |
| Date of interview |  |
| Time started |  |
| Time Finished |  |
| Rated name |  |

| **Score summary** 1. |
| --- |
|  |
| 2. |
|  |
| 3. |
|  |
| 4. |
|  |
| 5. |
|  |
| 6. |
|  |
| 7. |
|  |
| 8. (mean) |
|  |
| 9. (mean) |
|  |
| 10. |
|  |
| 11. |
| Total |
|  |

**Appendix 2: Insight Self Report (Insight SR)**

**1.I consider my anxiety to be an illness/disorder**

1. Most likely
2. Likely
3. Unlikely
4. Very Unlikely

**2. I consider my anxiety to be a psychological problem**

1. Most likely
2. Likely
3. Unlikely
4. Very Unlikely

**3. I consider my anxiety to be a normal reaction**

1. Most likely
2. Likely
3. Unlikely
4. Very Unlikely

**4. I believe that anxiety**

                                                                             Yes                          No                         Maybe

a) is an inherited condition

b) is caused by stress

c) is related to childhood experiences

d) is a chemical imbalance

e) is part of one’s personality

f) is part of life

g) is a psychiatric/psychological disorder

h) is curable

**5.I believe that taking prescribed medications can help me with my anxiety**

Strongly agree

Agree

Disagree

Strongly disagree

**6. I believe that psychological therapy can help me with my anxiety is with**

Strongly agree

Agree

Disagree

Strongly disagree

**7. I believe that a combination of prescribed medication and psychological therapy can help me with my anxiety**

Strongly agree

Agree

Disagree

Strongly disagree

**8. I believe that self-help/talking to people I trust can help me with my anxiety**

Strongly agree

Agree

Disagree

Strongly disagree

**9. I believe that diet and/or exercise can help me with my anxiety**

Strongly agree

Agree

Disagree

Strongly disagree

**10. I believe that carrying on as normal/ignoring it can help me with my anxiety**

 Strongly agree

Agree

Disagree

Strongly disagree

**Appendix 3: ­Internal Consistency, interrater and test-retest reliability of SAI-A items**

| SAI-A items | Mean (SD) | Corrected Item-total correlation | Alpha if item deleted | | Interrater ICC | Test-retest ICC | |  |
| --- | --- | --- | --- | --- | --- | --- | --- | --- |
| 1. Awareness of psychological changes | 1.37 (0.53) | 0.50 0.68 | |  | 0.76 | | 0.50 | |
| 2. Awareness of anxiety and irrational fear or worries | 1.50 (0.80) | 0.46 0.68 | |  | 0.59 | | 0.58 | |
| 3. Awareness of psychological condition | 0.87 (0.88) | 0.67 0.66 | |  | 0.67 | | 0.73 | |
| 4. Recognition of mental disorder | 0.76 (0.85) | 0.60 0.67 | |  | 0.62 | | 0.72 | |
| 5. Attribution of condition to mental disorder | 1.02 (0.93) | 0.45 0.68 | |  | 0.33 | | 0.97 | |
| 6. Awareness of consequences | 1.63 (0.77) | 0.46 0.68 | |  | 0.73 | | 1.00 | |
| 7. Awareness of needs for treatment | 1.32 (0.90) | 0.56 0.67 | |  | 0.66 | | 0.90 | |
| 8. Recognition of additional symptoms | 1.96 (0.91) | 0.22 0.70 | |  | 0.77 | | 0.69 | |
| 9. Attributions of symptoms to mental disorder | 0.41 (0.71) | 0.67 0.67 | |  | 0.68 | | 0.51 | |
| 10. Hypothetical contradiction | 1.33 (0.67) | 0.34 0.70 | |  | 0.40 | | 0.89 | |
| 11. Treatment engagement | 3.08 (1.11) | 0.27 0.70 | |  | 0.95 | | 0.86 | |
